# Identification and Molecular Docking Study of a Novel Angiotensin-I Converting Enzyme Inhibitory Peptide Derived from Enzymatic Hydrolysates of *Cyclina sinensis*

**DOI:** 10.3390/md16110411

**Published:** 2018-10-27

**Authors:** Fangmiao Yu, Zhuangwei Zhang, Liwang Luo, Junxiang Zhu, Fangfang Huang, Zuisu Yang, Yunping Tang, Guofang Ding

**Affiliations:** 1 Zhejiang Provincial Engineering Technology Research Center of Marine Biomedical Products, School of Food and Pharmacy, Zhejiang Ocean University, Zhoushan 316022, China; all_zzw@163.com (Z.Z.); luoliwang90@163.com (L.L.); gracegang@126.com (F.H.); abc1967@126.com (Z.Y.); tangyunping1985@163.com (Y.T.); 2 Laboratory of Aquatic Products Processing and Quality Safety, Marine Fisheries Research Institute of Zhejiang, Zhoushan 316021, China; zhujunxiang89@zjou.edu.cn

**Keywords:** *cyclina sinensis*, hypertension, ACE inhibitory peptides, inhibitory pattern, molecular docking

## Abstract

Marine-derived angiotensin-I converting enzyme (ACE) inhibitory peptides have shown potent ACE inhibitory activity with no side effects. In this study, we reported the discovery of a novel ACE-inhibitory peptide derived from trypsin hydrolysates of *Cyclina sinensis* (CSH). CSH was separated into four different molecular weight (MW) fractions by ultrafiltration. Fraction CSH-I showed the strongest ACE inhibitory activity. A peptide was purified by fast protein liquid chromatography (FPLC) and reversed-phase high-performance liquid chromatography (RP-HPLC) and its sequence was determined to be Trp-Pro-Met-Gly-Phe (WPMGF, 636.75 Da). The Lineweaver-Burk plot showed that WPMGF was a competitive inhibitor of ACE. WPMGF showed a significant degree of stability at varying temperatures, pH, and simulated gastrointestinal environment conditions. We investigated the interaction between this pentapeptide and ACE by means of a flexible molecular docking tool. The results revealed that effective interaction between WPMGF and ACE occurred mainly through hydrogen bonding, hydrophobic interactions, and coordination bonds between WPMGF and Zn(II). In conclusion, our study indicates that a purified extract derived from *Cyclina sinensis* or the WPMGF peptide could potentially be incorporated in antihypertensive functional foods or dietary supplements.

## 1. Introduction

Hypertension is the most common chronic disease and a critical factor in the development of cardiovascular pathologies. The number of yearly deaths due to hypertension complications is approximately 9.4 million worldwide [1], accounting for approximately 55% of all cardiovascular disease deaths [2]. Non-pharmaceutical approaches, including maintaining a healthy lifestyle and diet, not only prevent or delay the onset of hypertension, but also increase the efficacy of antihypertensive drugs, thereby reducing the risk of cardiovascular diseases [3].

Angiotensin-I converting enzyme (dipeptidyl carboxypeptidase, EC 3.4.15.1) belongs to the zinc-binding protease family [4]. This enzyme increases the production of angiotensin II by the renin-angiotensin system (RAS) [5], decreases the level of bradykinin produced by the kallikrein-kinin system (KKS) [6], and plays a critical physiological role in the regulation of peripheral blood pressure and electrolyte homeostasis. Therefore, a key to the treatment and prevention of hypertension is the effective inhibition of ACE activity [7].

Synthetic drugs such as captopril, enalapril, benazepril, and other ACE inhibitors [8] have been used clinically to prevent and treat hypertension. However, these synthetic inhibitors are associated with some clinically undesirable side effects, such as cough, taste disorders, angioedema, rash, and impaired renal function [9,10]. Therefore, investigators are searching for new ACE inhibitors with higher efficacy and fewer side effects. In view of the role of dietary therapy in the prevention and treatment of diseases, hydrolysates or peptides are being considered as key ingredients of functional foods or dietary supplements with antihypertensive activity. Although these require higher doses than synthetic antihypertensive drugs, they do not produce side effects [11].

Marine species account for about half of the world’s total biodiversity. This diversity, together with the availability of marine resources, have driven the search for new antihypertensive agents, which could be administered as functional foods or dietary supplements. Since the first discovery of a marine-derived peptide with ACE-inhibitory activity, researchers have identified a variety of antihypertensive peptides in hydrolysates from marine species, such as *Synodus macrops* [12] (AGPPGSDGQPGAK, IC_50_ = 0.42 mM), *Salmo salar* [13] (GAR and IGPR, IC_50_ = 0.598, 0.43 mM), shrimps (SSSKAKKMP, IC_50_ = 0.88 mM) [14], and *Ruditapes philippinarum* (pRPH, tRPH and nRPH, IC_50_ = 0.42, 2.93 and 3.53 mg/mL) [15]. Some marine-derived peptides are currently being evaluated for safety and efficacy through ongoing clinical trials which have advanced to different phases, and even a small number have entered the market. In 1999, *katsuobushi* oligopeptide was approved by the Japanese Ministry of Health and Welfare [16], and a linear pentapeptide derived from dried squid (*katsuobushi*, is one of the most common condiments used in Japanese cuisine) was incorporated into a blood pressure-lowering capsule and sold as a nutraceutical [17]. *Cyclina sinensis* is a bivalve mollusk belonging to the class Lamellibranchia, order Veneroida, family Veneridae [18]. It has been demonstrated to have a high protein content and a low-fat content, and the ratio of essential amino acids to total amino acids is about 45%. It has also been shown to be an ideal source of bioactive peptides with anti-tumor [19,20], anti-oxidative [19,21,22], and immunomodulatory activities [23,24]. Although marine-derived antihypertensive peptides from a variety of sources have been extensively investigated, ACE inhibitory peptides derived from *Cyclina sinensis* hydrolysates have seldom been reported.

For bioactive peptides to remain active after ingestion, they must be resistant to the gastrointestinal tract conditions [25]. In vitro methods offer a simpler way of investigating this issue than expensive, cumbersome animal experiments or in vivo tests, and yield detailed information. In the present study, trypsin was used to hydrolyze *Cyclina sinensis,* obtaining a peptide mixture, and the highest inhibitory activity peptides were purified by ultrafiltration (UF), fast protein liquid chromatography (FPLC), and reversed-phase high-performance liquid chromatography (RP-HPLC). The sequence of a purified peptide was determined by automatic Edman degradation with a PPSQ-31A protein sequencer, and its inhibitory characteristics were studied by means of Lineweaver-Burk plots. The effects of pH, heat, which mimic the gastroenteric environment on the ACE inhibitory activity of the purified peptide were also investigated. Furthermore, the interaction of the peptide with ACE was also investigated by molecular docking simulations.

## 2. Results and Discussion

### 2.1. Isolation and Purification of an Angiotensin-I Converting Enzyme (ACE) Inhibitory Peptide

**Figure 1:**
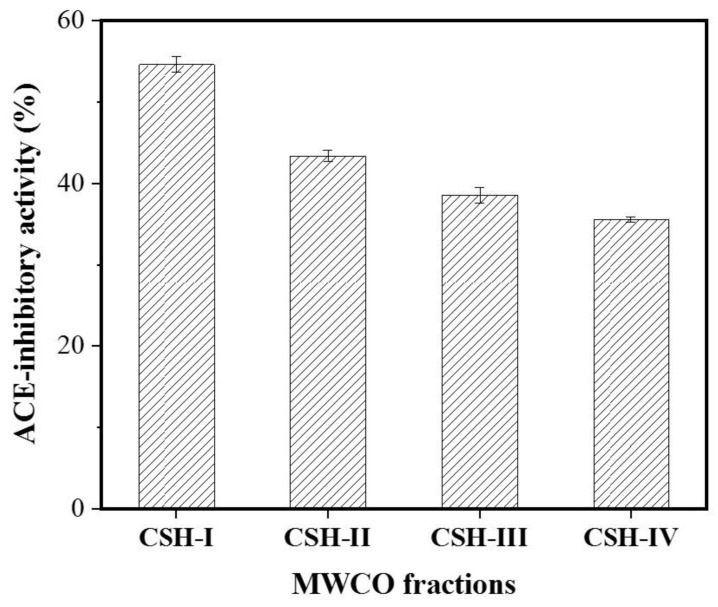
ACE inhibitory activity of hydrolysates from *Cyclina sinensis*. The *Cyclina sinensis* hydrolysates were divided into four parts by ultrafiltration: The MW of fraction CSH-I is less than 3 kDa; The MW of the fraction CSH-II is between 3 and 5 kDa; The MW of the fraction CSH-III is between 5 and 8 kDa; The MW of the fraction CSH-IV is above 8 kDa.

The molecular weight (MW) of a bioactive peptide determines to some extent whether the obtained bioactive peptide has the desired functional properties [26]. Ultrafiltration can be used to fractionate and partially purify protein hydrolysates to obtain bioactive peptides with both the desired MW and specific functions [27]. The CSH extract was pre-filtered with a 0.45 μm microporous membrane and was then separated by ultrafiltration into CSH-I (MW < 3 kDa), CSH-II (3 < MW < 5 kDa), CSH-III (5 < MW < 8 kDa) and CSH-IV (MW > 8 kDa) fractions. The ACE inhibitory activity of the fractions varied with their MW range. Among all the fractions, CSH-I (containing the lowest MW peptides) exhibited the strongest ACE inhibitory activity, with an inhibitory rate of 54.55%. In contrast, fractions containing peptides with a MW above 3 kDa possessed lower ACE inhibitory activity (Figure 1). Other studies have reported that low MW peptides are more active than high MW peptides because they can more readily enter the ACE active site, thereby inhibiting its catalytic activity [28,29]. Therefore, CSH-I was chosen for further purification.

**Figure 2:**
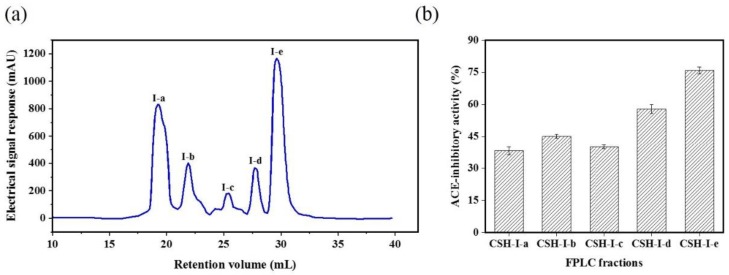
ACE inhibitory activity of fraction CSH-I from fast protein liquid chromatography (FPLC). (**a**) The fraction CSH-I was divided into five parts by AKTA-FPLC: I-a, I-b, I-c, I-d, I-e. (**b**) ACE inhibitory activity of AKTA-FPLC fractions from CSH-I.

**Figure 3:**
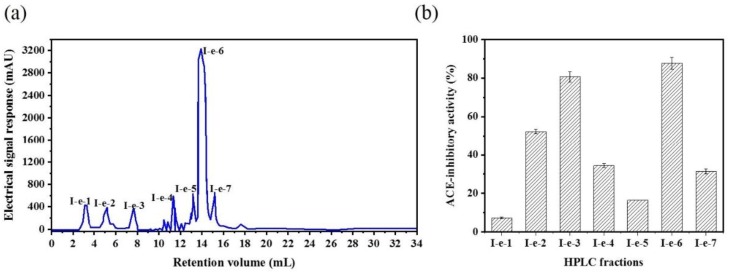
ACE inhibitory activity of fraction I-e from RP-HPLC. (**a**) The fraction CSH-I was divided into seven parts by RP-HPLC: I-e-1, I-e-2, I-e-3, I-e-4, I-e-5, I-e-6, I-e-7. (**b**) ACE inhibitory activity of RP-HPLC fractions from I-e.

**Figure 4:**
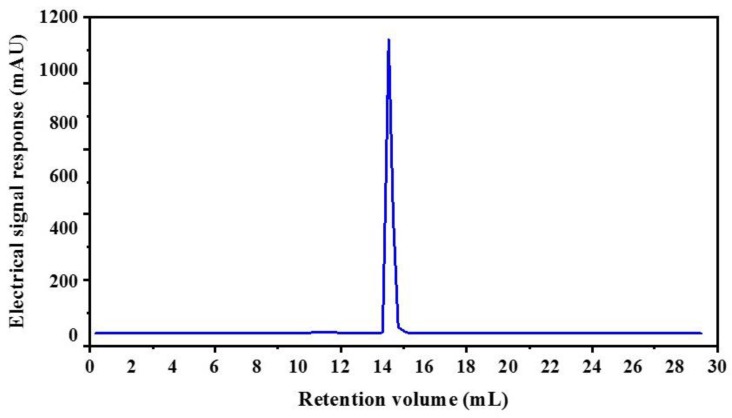
Purity analyze of I-e-6 fraction by RP-HPLC.

The CSH-I fraction was separated by means of an AKTA-FPLC system with a Superose^®^ (GE Healthcare, Chicago, IL, USA)12 10/300 GL agarose gel column into five fractions: I-a, I-b, I-c, I-d, I-e. As shown in Figure 2, there were five peptide peaks detected at a wavelength of 280 nm, and the five fractions associated with the peaks were separately pooled and freeze-dried to conduct ACE inhibitory activity tests. At a concentration of 1 mg/mL, all fractions showed inhibitory activity against ACE, but fraction I-e exhibited the strongest ACE inhibitory activity: 75.88%. This potent I-e fraction was further separated by means of RP-HPLC. We employed a ZORBAX SB-C_18_ analytical column and a linear gradient of acetonitrile (20% for 5 min, 100% for 30 min) containing 0.05% trifluoroacetic acid (TFA) to identify seven major absorbance peaks at 214 and 280 nm. Each fraction (I-e-1–I-e-7) was pooled and freeze-dried for additional ACE inhibitory activity tests. Fraction I-e-6 evidenced the most potent ACE inhibitory activity, with an inhibitory rate of 87.60% (Figure 3). The purity of fraction I-e-6 was then further increased by RP-HPLC. The chromatogram showed a major peptide peak with a retention volume of 14.16 mL, indicating that fraction I-e-6 had a satisfactory level of purity (Figure 4).

### 2.2. Amino Acid Sequence and IC_50_ Value of the Purified Peptide

**Figure 5:**
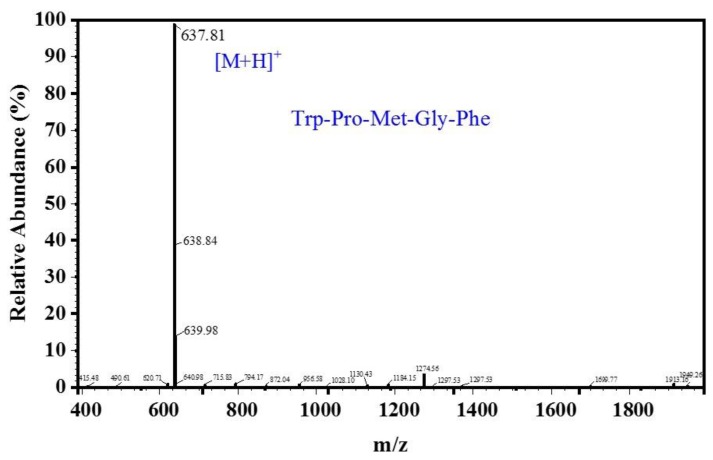
ESI-MS spectrum of the purified peptides.

As shown in Figure 5, the MW of the peptide, deduced from the m/z value of the quasi-molecular ion [M + H]^+^, was 637.81 Da. The amino acid sequence of the purified peptide, according to Edman degradation experiments and ESI-MS for amino acid sequence determination, was determined to be Trp-Pro-Met-Gly-Phe (WPMGF), and its theoretical MW was about 636.75 Da. To verify that the purified peptide had in vitro ACE-inhibitory activity, we obtained a synthetic peptide with the same pentapeptide sequence. This synthetic peptide showed identical ACE inhibitory activity as the pentapeptide purified from CSH, and its IC_50_ value was 0.789 mM.

According to previous reports, ACE inhibitory peptides are generally 2–16 amino acids long [30,31,32], although inhibitory activity has been detected in peptides up to 81 amino acids long [33]. Nevertheless, the structure–activity relationship of ACE inhibitory peptides is still not completely understood. ACE inhibitory peptides typically contain hydrophobic or aromatic amino acid residues at the C-terminus, such as Trp, Tyr, Pro, or Phe [34,35,36,37,38]. It has also been reported that the presence of N-terminal hydrophobic amino acid residues (such as Pro, Phe, Trp, or Met) also has a positive effect on their ACE inhibitory activity [39,40]. Hydrophobic amino acid residues decrease the solubility of antihypertensive peptides in the aqueous phase, but they also increase their solubility in a lipid environment (e.g., in the cell membrane), promoting greater antihypertensive effects [41]. According to the Edman degradation experiments, the peptide we identified (WPMGF) has hydrophobic amino acid residues at both the C-terminus and N-terminus.

### 2.3. Analysis of the ACE Inhibitory Characteristics of Peptide WPMGF

**Figure 6:**
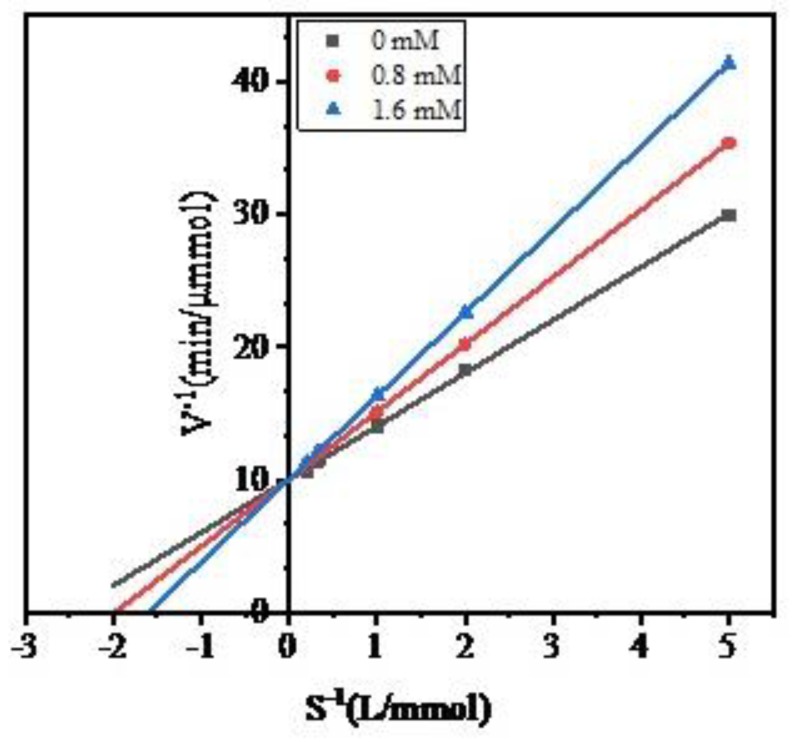
The Lineweaver-Burk plot for the ACE inhibition pattern of purified peptide.

To investigate the inhibitory characteristics of peptide WPMGF, the *S*^−1^ concentration of the ACE synthetic substrate hippuryl-l-histidyl-l-leucine (HHL) was plotted against the rate of formation (*V*^−1^) of hippuric acid (HA). The Lineweaver–Burk plots are shown in Figure 6. In the Lineweaver-Burk equation, *K_i_* is the x-intercept, 1/*V_max_* is the y-intercept, and *K_m_*/*V_max_* is the slope [42]. The Lineweaver-Burk plots of the control (uninhibited) enzyme and of the enzyme in the presence of varying concentrations of peptide WPMGF have the same y-intercepts. This indicates that the Michaelis-Menten constant (*K_m_*) increases while *V_max_* remains unchanged, a distinguishing feature of competitive inhibitors. We calculated the K_i_ from the Lineweaver–Burk plots, which were −1.98 and −1.60 at 0.8 mM and 1.6 mM, respectively. Thus, WPMGF is a competitive inhibitor of ACE. Competitive inhibitors can compete with the substrate for the catalytic site of the enzyme or can alter the conformation of the enzyme, ultimately inhibiting its activity [43]. In recent years, many competitive ACE inhibitory peptides have been reported, such as YQK (Tyr-Gln-Lys) from bovine casein [44], RYL (Arg-Tyr-Leu) from Silkworm Pupa [45], VVSLSIPR (Val-Val-Ser-Leu-Ser-Ile-Pro-Arg) from pigeon pea (*Cajanus cajan*) [46], and YLYELAR (Tyr-Leu-Tyr-Glu-Leu-Ala-Arg), AFPYYGHHLG (Ala-Phe-Pro-Tyr-Tyr-Gly-His-His-Leu-Gly) from scorpion (*Hemiscorpius lepturus*) venom. In addition, commercial functional foods or dietary supplements [16,17], as well as many antihypertensive drugs [47], have also been reported to be competitive inhibitors of ACE. Some studies have reported that optimal competitive inhibition is associated with the presence of hydrophobic amino acid residues at both the C-terminus and N-terminus of the peptides [48].

### 2.4. Stability of Peptide WPMGF

**Figure 7:**
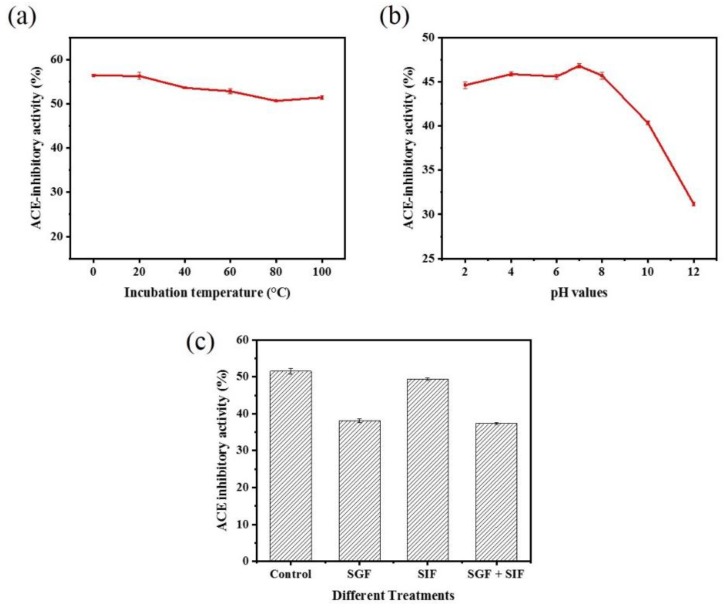
Stability of ACE inhibitory activity of peptide WPMGF. (**a**) Stability of ACE inhibitory activity of peptide WPMGF after 2 h of treatment at different temperatures (0, 20, 40, 60, 80, and 100 °C); (**b**) Stability of ACE inhibitory activity of peptide WPMGF after 2 h of treatment at different pH values (2, 4, 6, 8, 10 and 12); The investigation of thermal stability and pH stability were carried out in 0.1 M borate buffer containing the peptide WPMGF at 0.5 mg/mL. (**c**) Stability of ACE inhibitory activity of peptide WPMGF after digestion with SGF_[sp]_ and SIF_[sp]_. Control: peptide WPMGF; SGF: peptides were digested with SGF_[sp]_ for 2 h; SIF: peptides were digested with SIF_[sp]_ for 2 h; SGF + SIF: peptides were successively digested with SGF_[sp]_ for 2 h and SIF_[sp]_ for 2 h; The investigation of simulated gastroenteric environments were carried out in SGF_[sp]_ or SIF_[sp]_ containing the peptide WPMGF at 0.5 mg/mL.

When peptides are used as functional food ingredients or food additives, they must show stability after heat treatment, and this is a prerequisite for their large-scale industrial development and production [49]. Our results showed that moderate temperatures (below 40 °C) did not significantly affect the ACE inhibitory activity of peptide WPMGF (Figure 7a). The inhibitory effect decreased slightly after heating the peptide at 100 °C for 2 h. However, it is important to point out that these are extreme conditions, indicating that WPMGF maintained a significant degree of stability after extreme and prolonged heat treatment. These stability results were similar to those reported with Lizardfish (*Synodus macrops*) scale gelatin peptides heated at 100 °C for 2 h [12]. In contrast, lower temperatures did decrease the ACE inhibitory activity of peptide WPMGF, reaching only 46% at 0 °C, a decrease of about 10 points. These data indicate that peptide WPMGF is stable when the temperature remains at or above body temperature, a characteristic that is desirable for the industrial production of antihypertensive functional foods or dietary supplements [50]. This result is also consistent with the reported thermal stability of an ACE inhibitory peptide derived from a 10 kDa fraction of soybean milk fermented by *Lactobacillus plantarum* strain C2 [51], which maintains almost the same inhibitory activity after various thermal treatments (25–100 °C, 2 h).

Bioactive peptides encounter different pH levels during their transit through the gastrointestinal tract, so it is important to test their stability under these conditions. The ACE inhibitory activity of peptide WPMGF decreased slightly in a strong acidic environment (pH 2, 4). There were no significant differences in the ACE inhibitory activity after exposure to a weak acid (pH 6) or a weak base (pH 8) environment when compared to pH 7. In contrast, activity was significantly reduced at pH 10 and 12. (Figure 7b). The low pH of gastric juice is one of the main reasons why a bioactive peptide can lose activity before it is absorbed in the intestinal tract to exert specific physiological functions [51]. The pH of human gastric juice ranges from 2 to 5, but during the intestinal digestion phase the pH is almost neutral [52]. It is significant that the inhibitory activity of peptide WPMGF from *Cyclina sinensis* was maintained at pH 2–8. This indicated that the ACE inhibitory peptide showed a satisfactory degree of pH stability, conducive to it resisting the gastrointestinal digestion and production processes. Other studies have reported that peptides retain their ACE inhibitory activity after various pH treatments [44]. The loss of activity at pH 10, 12 may be due to hydrolysis of the purified peptide under extremely alkaline conditions, affecting its structure, amino acid composition, and hydrophobicity. However, this would not affect the possible applicability proposed in this paper.

Bioactive peptides must either remain stable after the gastrointestinal digestion process and retain their biological activity after passing through the intestinal wall, or be converted to other active forms after digestion [53]. For example, it has been reported that after oral administration, some peptides with ACE inhibitory activity do not show the expected hypotensive effects because these peptides are hydrolyzed by gastrointestinal proteases [54]. An in vitro simulated gastrointestinal environment provides a simple method to evaluate changes in the ACE inhibitory activity of peptides after oral administration. To evaluate the stability of peptide WPMGF under simulated gastrointestinal conditions, we incubated it with simulated gastric fluid, SGF_[sp]_, and simulated intestinal fluid, SIF_[sp]_. The results showed that the ACE inhibitory activities of peptide WPMGF after incubation with SGF_[sp]_ and SIF_[sp]_ were 38.16 ± 0.52% and 49.40 ± 0.30%, respectively, whilst that of the control was 51.52 ± 0.72% (Figure 7c). Significant changes in the ACE inhibitory activity occurred after incubation with SGF_[sp]_. Pepsin hydrolyzes mainly carboxy terminal peptide bonds containing hydrophobic amino acid residues such as Phe, Trp, and Tyr, and the peptide we identified has the sequence Trp-Pro-Met-Gly-Phe. Thus, pepsin in gastric juice may be hydrolyzing the active fragment of the partially purified peptide or partially degrading it into smaller peptide fragments. In this regard, several reports have shown that ACE inhibitory peptides are susceptible to degradation by pepsin in vitro [55,56]. In contrast, no significant changes in ACE inhibitory activity occurred after incubation with SIF_[sp]_, indicating that peptide WPMGF may be resistant to digestion in the intestinal tract. However, since the active sequence of the peptide was not determined, it cannot be concluded that peptide WPMGF remained intact.

Veronique Schulten et al. established a model for studying the in vivo absorption of proteins in the absence or presence of a food matrix [57]. The results showed that the presence of a protein-rich food matrix significantly delayed the gastrointestinal digestion of food allergens and enhanced their stability under simulated gastrointestinal conditions. Based on this study, we speculate that food carbohydrate and proteins can compete with bioactive peptides for enzymatic cleavage sites, delaying their gastrointestinal digestion. In addition, polysaccharide components may reduce the activity of gastric enzymes, thereby enhancing the stability of bioactive peptides [58,59,60]. Therefore, if peptide WPMGF were to be included in antihypertensive functional foods, certain food matrix components could be added to promote its stability under gastrointestinal digestive conditions.

### 2.5. Insights into the Molecular Docking Mechanism

**Figure 8:**
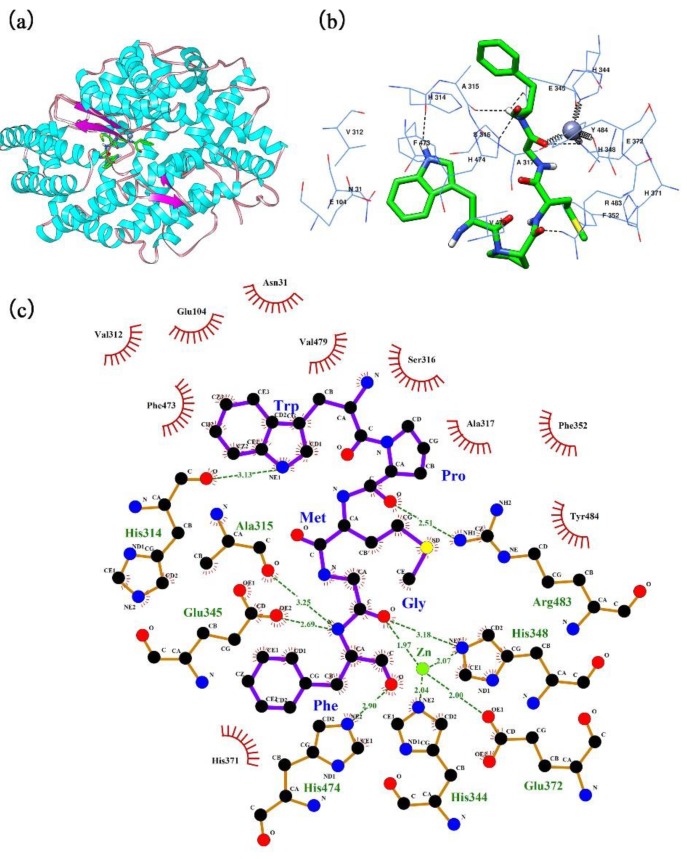
The best-ranked docking pose of WPMGF binding with ACE (PDB: 1O86). (**a**) General overview of docking pose of WPMGF (green stick model) at the ACE active site. (**b**) Local overview of docking pose of WPMGF (green stick model) at the ACE active site. (**c**) The binding mode between ACE residues and WPMGF (green stick model) after docking at the ACE active site. Green dotted line indicates the formation of molecular forces, and the color of the specific molecular force were not distinguished.

The automated docking tool of the AutoDock 4.2 software was used to simulate the molecular interaction between ACE and the pentapeptide isolated from *Cyclina sinensis*. A comprehensive view of this interaction in the presence of the Zn(II) prosthetic group and with a binding energy value of −29.97 kJ/mol (Figure S1) is shown in Figure 8. The optimal docking pose of the pentapeptide in relation to ACE is shown in Figure 8b,c. WPMGF was deeply buried in the catalytic active site of ACE and was tightly surrounded by amino acid residues to form a stable ACE-pentapeptide complex. In the docking conformation, with WPMGF as a ligand, and ACE as a donor, three types of molecular forces participated: Electrostatics interactions, hydrogen bonds, and hydrophobic interactions. Although most of the interactions were mediated by hydrogen bonds, there were many hydrophobic interactions between peptide WPMGF and ACE, including 10 amino acid residues in ACE, i.e., Val312, Glu104, Asn31, Phe473, Val479, Ser316, Ala317, Phe352, Tyr484, and His371. These hydrophobic interactions facilitate the encapsulation of the peptide in the catalytic cavity of ACE, promoting efficient binding to the ACE active site. In the best docking pose simulation, abundant hydrogen bond forces were also generated between ACE residues and the pentapeptide, which may explain the binding energy of up to −29.97 kJ/mol (Table S1). WPMGF formed hydrogen bonds with ACE residues S1 pocket (Ala315, Glu345), S2 pocket (His314, His474), Arg483, and His348, a pattern that is extremely similar to the hydrogen bonds formed in the lisinopril-ACE complex. The presence of Zn(II) in the enzyme active center played a critical role in the ACE inhibitory activity, since amino acid residues His348, Glu372, His344 of the ACE formed coordinate bonds with the Zn(II) prosthetic group. This may explain the deformation of the Zn(II) tetrahedral coordination and accelerated deactivation of ACE [38].

## 3. Materials and Methods

### 3.1. Materials and Chemicals

*Cyclina sinensis* was purchased at the Dinghai Nanzhen fish market in Zhoushan City, China. After being positively identified by Professor Zhao Shenglong of Zhejiang Ocean University, the samples were shipped in ice to our biology laboratory within 30 minutes of purchase. *Cyclina sinensis* was cleaned, dehulled, washed with pure water, and then homogenized with a grinder (5000 rpm, 5 min). The tissue homogenate was incubated 3 times with 0.1 M NaOH, and each incubation lasted 2 h, then filtered with a gauze. The filter was then washed with water until the pH reached neutrality, and the homogenate was dehydrated and stored in sealed bags at −20 °C until the next separation and purification steps.

Trypsin (from bovine pancreas, 200 U/mg) and pepsin (from porcine gastric mucosa, 300 U/mg) were purchased from YTHX Biotechnology Co., Ltd. (Beijing, China). ACE (from rabbit lung), and the ACE synthetic substrate hippuryl-l-histidyl-l-leucine (HHL) were purchased from Sigma Chemicals Co. (St. Louis, MO, USA). Other chemicals and reagents used were of analytical grade, except acetonitrile and trifluoroacetic acid (TFA), which were HPLC grade (Sinopharm Chemical Reagent Co., Ltd, Shanghai, China).

### 3.2. Production of Cyclina Sinensis (CSH)

The pretreated *Cyclina sinensis* tissue homogenate was subjected to hydrolysis with trypsin under the conditions reported by Luo et al. [61] (Solid-liquid ratio 1 g:2 mL, enzyme concentration 1200 U/g, pH 8.0, hydrolysis at 45 °C for 9 h). The pH of the reaction mixture was maintained at the desired value by intermittently adding a small volume of 0.5 M NaOH or 0.5 M HCl by means of a constant flow pump. After 9 h of hydrolysis with trypsin, the enzymatic reaction was terminated by heating the reaction mixture until it boiled. The enzymatically hydrolyzed *Cyclina sinensis* homogenate was then centrifuged at 10000 rpm for 20 min to separate the CSH from the insoluble material. Finally, CSH was vacuum freeze-dried with an Alpha 1–4 LD plus freeze dryer (Marin Christ, Osterode, Germany), collected in sealed penicillin bottles and stored at 20 °C.

### 3.3. Determination of ACE Inhibitory Activity

To determine the ACE inhibitory activity, we used the method described by D.W.Cushman [62] and Lisete Paiva [63], with slight modifications. Briefly, 120 μL of 5 mM HHL solution and 20 μL of 1 mg/mL sample solution were mixed thoroughly and pre-incubated for 5 min at 37.5 °C. The control was prepared by replacing the sample solution with 20 μL of 300 mM NaCl in 100 mM borate buffer (pH 8.3). Then, 10 μL of ACE (0.1 U/mL) solution was added, tubes were mixed well, and incubated at 37.5 °C for 1 h in a water bath. The reaction was terminated by the addition of 150 μL of 1 M HCl. The reaction solutions were then filtered through a 0.45 μm microporous membrane and quantified by using a ZORBAX SB-C_18_ analytical column (4.6 mm × 250 mm, 5 μm, 25 °C) and an Agilent HPLC ChemStation, consisting of a G1311C quaternary pump VL, a G1329B standard autosampler, a G1316A column oven, and a G1314F variable wavelength detector. The eluate was acetonitrile-ultra-pure water (25:75, *v*/*v*) containing 0.05% trifluoroacetic acid, and the 10 μL injection was eluted at a flow rate of 0.8 mL/min. Hippuric acid (HA) was detected at 228 nm. The average value from three separate measurements was used to calculate the ACE inhibition rate according to the following equation:

(1) $$\mathrm{ACE}\;\mathrm{inhibition}\%=\frac{\left(B-S\right)}{B}\times 100$$

where *B* is the HA peak area in the normal group (mAU.s) and *S* is the HA peak area in the sample group (mAU.s). The IC_50_ value is defined as the concentration required to inhibit 50% of ACE activity.

### 3.4. Isolation and Purification of ACE-Inhibitory Peptides

#### 3.4.1. Ultrafiltration (UF)

Fractions of different MW were separated by ultrafiltration membrane technology in tangential flow mode. After preliminary filtration through a 0.45 μm microporous membrane, the Cogent μScale TFF ultrafiltration system (Merck Millipore, Jaffrey, NH, USA) with MW cut-off membranes of 8, 5 and 3 kDa was used to separate the CSH fractions. Fractions were pooled, vacuum freeze-dried, and then evaluated for their ACE-inhibitory activity.

#### 3.4.2. Fast protein liquid chromatography (FPLC)

The ACE inhibitory activity of the different fractions obtained in the previous step was compared, and the most active fraction was separated and purified with an AKTA-FPLC system (AKTA Purifier UPC 100, GE Healthcare, Uppsala, Sweden). The sample solution (0.1 mg/mL) was filtered through a 0.45 μm microporous membrane and 500 µL was injected into the AKTA-FPLC system equipped with a Superose^®^ 12 10/300 GL agarose gel column (10 mm × 300 mm, 10 μm). Elution was carried out at 0.5 mL/min with ultrapure water, fractions were detected at 280 nm, and collected automatically (3.5 mL/tube). The fractions corresponding to each absorption peak were pooled and freeze-dried, and their ACE inhibitory activity was determined.

#### 3.4.3. Reversed-phase high-performance liquid chromatography (RP-HPLC)

The fraction (500 μL) showing the most significant inhibition was further separated and purified by means of an Agilent HPLC ChemStation and a ZORBAX SB-C_18_ analytical column (4.6 mm × 250 mm, 5 μm) set at 25 °C. Mobile phase A consisted of 0.05% TFA in ultrapure water and mobile phase B was 0.05% TFA in acetonitrile. The fraction showing the most significant inhibitory activity was again assayed for purity by means of RP-HPLC to determine whether it was a single active fraction. The gradient initiated with 20% solvent B, increasing after 5 min to 100% solvent B, and remaining constant for 30 min, with a flow rate of 1.0 mL/min.

### 3.5. N-Terminal Sequencing of the ACE Inhibitory Peptide

The MW of the peptide was determined based on the increased proton [M + H]^+^ formed during mass spectrometry ionization, and the identification of the peptide sequence was performed by Scilongs Biotechnology Co., Ltd (Beijing, China). The amino acid sequence of the purified peptide was determined by the Edman chemical degradation technique using a PPSQ-31A protein sequencer (Shimadzu, Japan).

### 3.6. Synthesis of ACE Inhibitory Peptide

Any ACE inhibitory effects from other proteins can be excluded by testing a purified synthetic peptide, which can also be used in subsequent experiments. Mimotopes Biotechnology Co., Ltd. (Wuxi, China) synthesized the synthetic peptide using the Fmoc-solid phase method based on the sequence identified measured in the previous step. The identity of the peptide was verified by mass analysis using ESI-MS, and the purity of the synthetic peptide was detected above 95% by HPLC.

### 3.7. Determination of the ACE-Inhibitory Characteristics of the Purified Peptide

We followed the method described by Bita Forghani [64] to determine the ACE inhibitory pattern of the synthetic peptide, with minor modifications. Briefly, 0.5 mg/mL or 1 mg/mL of ACE inhibitory peptide solution was incubated with different concentrations of HHL (0.2, 0.5, 1.0, 3.0, or 5.0 mmol/L), and the ACE inhibitory activity was determined as described previously. The inhibitory pattern was determined by means of the Lineweaver-Burk Equation [65], plotting the concentration S^−1^ of HHL against the rate of formation V^−1^ of HA. The rate of HA generation was calculated based on the following equation:

(2) $$V=\frac{300\times C_{\mathrm{HA}}}{60}=5C_{\mathrm{HA}}$$

where V is the rate of formation of HA (μmol/min); 300 is the total volume of the ACE reaction solution (μL); 60 is the reaction time (min), and *C_HA_* is the concentration of HA (mmol/L).

### 3.8. Stability Studies

#### 3.8.1. Thermal and pH Stability of the ACE-Inhibitory Peptide

The method described by Kittiphattanabawon et al. [66] was used to determine the thermal and pH stability. The reactions were carried out in 0.1 M borate buffer containing the ACE inhibitory peptide at 0.5 mg/mL. Samples were incubated at different temperatures (0, 20, 40, 60, 80, and 100 °C) for 2 h. After samples returned to room temperature (25 °C), the pH of the solution was adjusted to neutral (pH = 7), and the ACE inhibitory activity was evaluated by HPLC. Samples were also incubated at pH 2, 4, 6, 8, 10, and 12 for 2 h at room temperature to evaluate the pH stability.

#### 3.8.2. Effects of Simulated Gastroenteric Environments on the ACE-Inhibitory Activity of the Peptide

We followed the methods described in the Pharmacopoeia of the People’s Republic of China 2015 Edition [67] (Volume IV, General requirements, Page 118):

Simulated gastric fluid (SGF_[sp]_): Concentrated hydrochloric acid (37%) was diluted to a 9.5% to 10.5% solution. Then, 800 mL of water and 10 g of pepsin were added to 16.4 mL of this diluted hydrochloric acid solution, mixed well, and the volume completed to 1000 mL with water.

Simulated intestinal fluid (SIF_[sp]_): 6.8 g of KH_2_PO_4_ was dissolved in 500 mL of water, and the pH was adjusted to 6.8 with 0.1 M NaOH. Then ten grams of trypsin were dissolved in an appropriate amount of water, added to the solution described above, and diluted to 1000 mL with water.

The in vitro stability of the ACE inhibitory peptide in the simulated gastrointestinal environments was determined according to the method described by Yu et al [68]. The most active peptide solutions were individually mixed with SGF_[sp]_ or SIF_[sp]_ (0.5 mg/mL) and incubated for 2 h at 37.5 °C. In addition, the peptide solution was first incubated with SGF_[sp]_ at 37.5 °C for 2 h, the solvent was removed by means of a centrifugal filter concentrator, and was then incubated with SIF_[sp]_ for 2 h. The reaction mixtures were then boiled to inactivate the enzyme. After cooling to room temperature, samples were centrifuged at −4 °C (10000 rpm, 15 min) and the supernatant was adjusted to pH = 7 for the determination of the ACE inhibitory activity.

### 3.9. Molecular Docking of the Peptide in the ACE Binding Site

The crystal structure of human ACE (PDB: 1O86) was obtained from the Research Collaboratory for Structural Bioinformatics (RCSB) Protein Data Bank (http://www.rcsb.org). Prior to docking analysis, water molecules and the Lisinopril inhibitor were removed using the Chimera 1.13 software (University of California, San Francisco, CA, USA) [69], whereas the zinc cofactor was retained in the ACE protein model. The structure of the ligand peptide was also determined using the Chimera 1.13 software. Then, the AutoDock 4.2 software [70] with free-energy scoring function was used to determine the possible protein–ligand complex conformation. The grid box (50 × 70 × 50 Å) was defined to cover all active residues around the Zn(II) prosthetic group. The best-ranked docking pose of the ligand peptide in relation to the target protein was determined based on the scores and binding-energy values. The Δ*G*_binding_ was computed according to the following equation:

(3) $$\Delta G_{\mathrm{binding}}=\Delta G_{\mathrm{vdW}+\mathrm{hb}+\mathrm{desolv}}+\Delta G_{\mathrm{elec}}+\Delta G_{\mathrm{intern}}+\Delta G_{\mathrm{tor}}-\Delta G_{\mathrm{unb}}$$

where, Δ*G*_vdW+hb+desolv_ is the sum of the van der Waals, hydrogen bond, and desolvation energies. Δ*G*_elec_ is the electrostatic energy, Δ*G*_intern_ is the total internal energy, Δ*G*_tor_ is the torsional free energy, and Δ*G*_unb_ is the unbound system’s energy.

### 3.10. Statistical Analysis

All experimental data were expressed as the mean ± standard deviation ($\bar{x}$ ± s, n = 3). Data were analyzed using SPSS software, version 24.0 (SPSS Inc., Chicago, IL, USA).

## 4. Conclusions

This study reports, for the first time, that trypsinized hydrolysates of *Cyclina sinensis* possess high ACE inhibitory activity. After a series of chromatographic separation and purification steps, a novel ACE inhibitory peptide was identified with the sequence Trp-Pro-Met-Gly-Phe (WPMGF; MW = 636.75 Da). The purified pentapeptide exhibited potent ACE inhibitory activity with an IC_50_ value of 0.789 mM, and its inhibitory pattern was shown to be of a competitive nature. WPMGF exhibited sustained thermal stability at 100 °C, maintained high inhibitory activity after being subjected to a wide range of pH values (2–8), and showed satisfactory residual activity after in vitro simulation of gastrointestinal digestive conditions. The peptide (WPMGF) has hydrophobic amino acid residues at both the C-terminus and N-terminus, a feature commonly found in peptides with good ACE inhibitory activity. Molecular docking simulation indicates that the peptide binds well to the active site of ACE and the conformation of the pentapeptide-ACE complex is stable. Therefore, this peptide derived from trypsinized hydrolysates of *Cyclina sinensis*, together with a certain proportion of food matrix components, could be considered in the future for the industrial production of functional foods and dietary supplements.

## Supplementary Materials

The following are available online at http://www.mdpi.com/1660-3397/16/11/411/s1, Figure S1: The calculation of binding energy between pentapeptide WPMGF and ACE, Table S1: All docking conformations of the pentapeptide WPMGF for ACE.
