# Supplementary material for: Identification and Molecular Docking Study of a Novel Angiotensin-I Converting Enzyme Inhibitory Peptide Derived from Enzymatic Hydrolysates of Cyclina sinensis
Source: Mar Drugs. 2018 Oct 27;16(11):411. doi: 10.3390/md16110411 (PMC6265983; doi:10.3390/md16110411)
Supplement: Supplementary file 1 [file marinedrugs-16-00411-s001.pdf]

Table S1. All docking conformations of the pentapeptide WPMGF for ACE.

Conformational ranking	Binding energy (KJ mol^{-1})	Ligand efficiency	Inhibition constant (μM)
1	−29.97	−0.16	5.57
2	−28.67	−0.16	9.34
3	−27.55	−0.15	14.78
4	−27.42	−0.15	15.47
5	−27.38	−0.15	15.89
6	−25.50	−0.14	33.88
7	−25.00	−0.14	41.66
8	−23.95	−0.13	62.63
9	−23.49	−0.13	76.36
10	−23.45	−0.13	77.63
11	−22.74	−0.12	102.21
12	−22.53	−0.12	112.27
13	−22.45	−0.12	115.24
14	−22.36	−0.12	119.94
15	−22.24	−0.12	126.08
16	−22.11	−0.12	132.18
17	−21.44	−0.12	173.57
18	−21.02	−0.11	204.35
19	−20.98	−0.11	207.72
20	−20.94	−0.11	212.18
21	−20.94	−0.11	213.30
22	−20.90	−0.11	217.71
23	−20.44	−0.11	261.03
24	−20.27	−0.11	278.45
25	−19.65	−0.11	360.41
26	−19.44	−0.11	390.42
27	−18.64	−0.10	541.06
28	−18.52	−0.10	566.35
29	−18.48	−0.10	572.59
30	−18.48	−0.10	574.95
31	−18.10	−0.10	675.09
32	−17.81	−0.10	749.26
33	−17.43	−0.09	874.01
34	−16.84	−0.09	1120
35	−16.47	−0.09	1280
36	−16.30	−0.09	1370
37	−15.84	−0.09	1670
38	−15.76	−0.09	1730
39	−15.26	−0.08	2110
40	−15.09	−0.08	2270
41	−13.75	−0.07	3900
42	−13.67	−0.07	4000
43	−13.33	−0.07	4610
44	−13.17	−0.07	4910
45	−11.24	−0.06	10610
46	−10.32	−0.06	15350
47	−10.07	−0.05	17100
48	−9.78	−0.05	19160
49	−9.66	−0.05	20350
50	−3.38	−0.02	253970

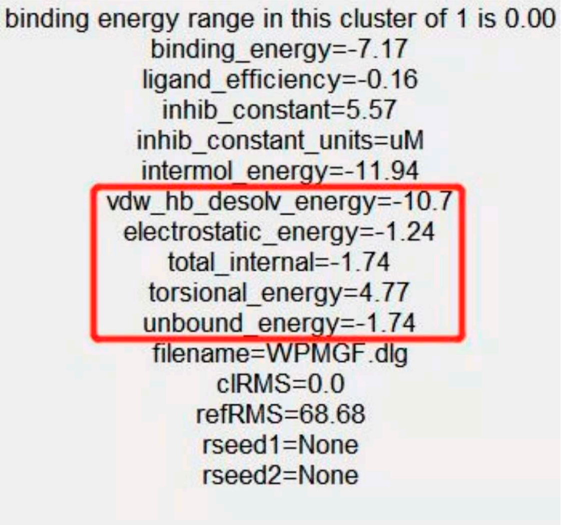

Figure S1. The calculation of binding energy between pentapeptide WPMGF and ACE

$$\Delta G_{\text{binding}} = \Delta G_{\text{vdW+hb+desolv}} + \Delta G_{\text{elec}} + \Delta G_{\text{intern}} + \Delta G_{\text{tor}} - \Delta G_{\text{unb}}$$

= -10.7-1.24-1.74+4.77-(-1.74) = -7.17 kcal/mol= -29.97 kJ/mol
